# Supplementary figures and images for: Genome-Wide Identification, Characterization and Experimental Expression Analysis of CNGC Gene Family in Gossypium
Source: Int J Mol Sci. 2023 Feb 27;24(5):4617. doi: 10.3390/ijms24054617 (PMC10003296; doi:10.3390/ijms24054617)

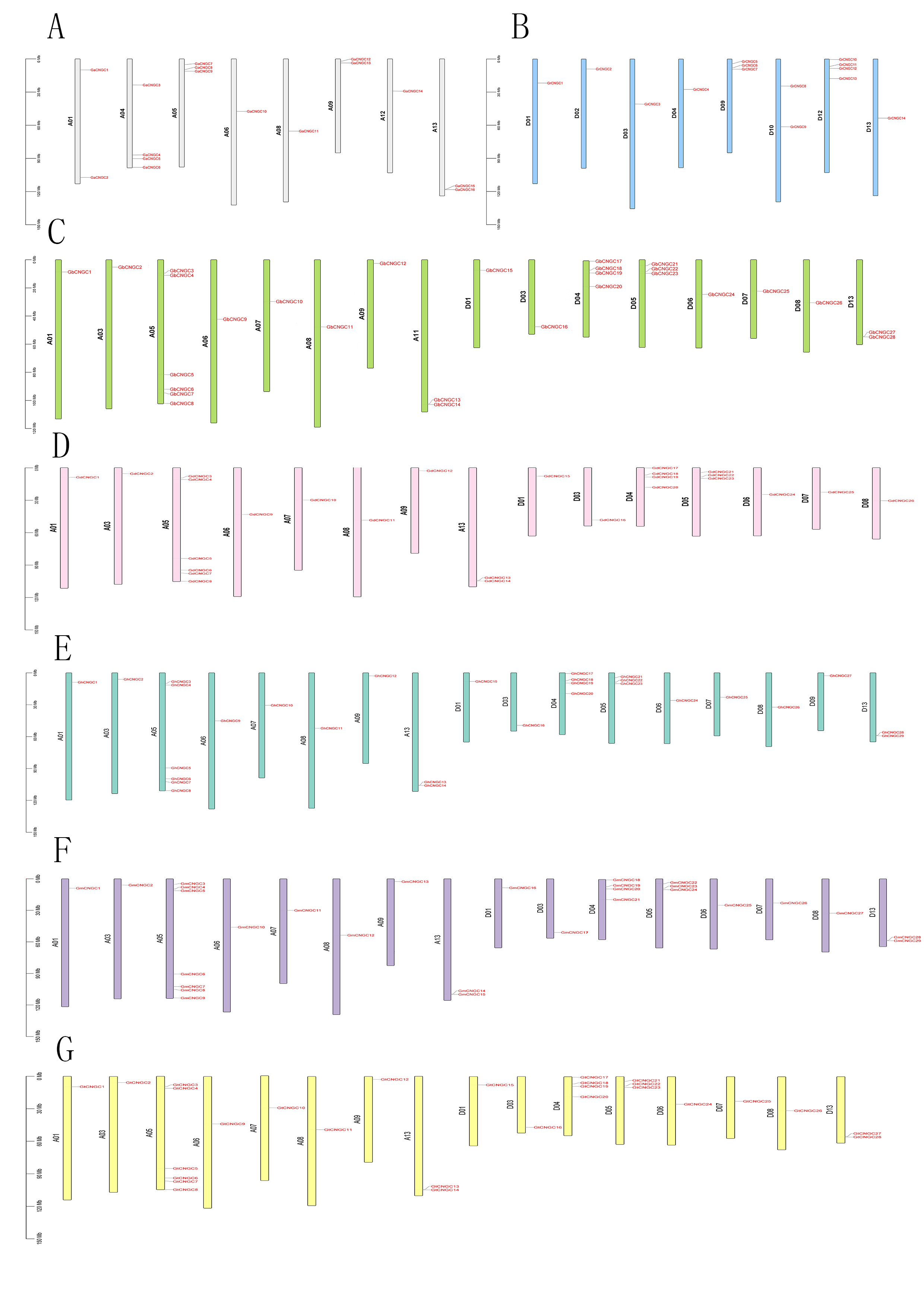

Supplement: Supplementary file 1 [file ijms-24-04617-s001.zip › Figure.S1.jpg]
